# Study of the Biologically Active Properties of Medicinal Plant *Cotinus coggygria*

**DOI:** 10.3390/plants10061224

**Published:** 2021-06-16

**Authors:** Stanislav Sukhikh, Svetlana Noskova, Artem Pungin, Svetlana Ivanova, Liubov Skrypnik, Evgeny Chupakhin, Olga Babich

**Affiliations:** 1 Institute of Living Systems, Immanuel Kant Baltic Federal University, A. Nevskogo Street 14, 236016 Kaliningrad, Russia; SSukhikh@kantiana.ru (S.S.); svykrum@mail.ru (S.N.); apungin@kantiana.ru (A.P.); LSkrypnik@kantiana.ru (L.S.); chupakhinevgen@gmail.com (E.C.); olich.43@mail.ru (O.B.); 2 Department of Bionanotechnology, Kemerovo State University, Krasnaya Street 6, 650043 Kemerovo, Russia; 3 Natural Nutraceutical Biotesting Laboratory, Kemerovo State University, Krasnaya Street 6, 650043 Kemerovo, Russia; 4 Department of General Mathematics and Informatics, Kemerovo State University, Krasnaya Street, 6, 650043 Kemerovo, Russia

**Keywords:** *Cotinus coggygria*, biologically active substances, extracts, toxicological, antimicrobial properties, antioxidant activity

## Abstract

The results of the studies have shown that to obtain an extract of a complex of biologically active substances of *Cotinus coggygria*, ethyl alcohol (mass fraction of alcohol 70%) with a hydromodule of 1:5 should be used, and the extraction should be carried out for 60 min at a temperature of 60 °C. The investigated plant extracts with the complex of bioactive substances from the *Cotinus coggygria* leaves and flowers are safe from the point of view of the content of heavy metals, pesticides, aflatoxin B1, radionuclides, as well as pathogenic and opportunistic microorganisms. It has been established that the *Cotinus coggygria* extract contains rutin, hyperoside, ferulic acid, quercetin, kaempferol, disulphuretin, sulphurein, sulphurein, gallic acid, methyl gallate, pentagalloyl glucose, 3,3′,4′,5,6,7-hexahydroxyflavonone, 3,3′,4′,5,5′,7-hexahydroxyflavonone, 3-*O*-α-L-rhamnofuranoside, 3,3′,4′,5,5′,7-hexahydroxyflavulium(1+), 7-*O*-β-D glucopyranoside, and 3,3′,4′,7-tetrahydroxyflavonone. The tested extracts have anticancer, antigenotoxic, and antimicrobial (against *E. coli*, *S. aureus*, *P. vulgaris*, *C. albicans*, *L. mesenteroides*) properties. The high antioxidant status of the tested extracts was established; the antioxidant activity of the samples was 145.09 mg AA/g (AA—ascorbic acid).

## 1. Introduction

All plants, including medicinal ones, synthesise and accumulate natural compounds of primary and secondary synthesis during their growth. The primary synthesis substances include compounds that perform energy and plastic functions—proteins, lipids, and carbohydrates. Secondary metabolites—iridoids, phytoncides, tannides, flavonoids, essential substances, nitrogen-containing compounds, glycosides, and organic acids—have unique antibacterial, antioxidant, and other properties that allow using medicinal plants in food, pharmaceutical, feed, and other industries [1,2].

Natural products produced by higher plants as secondary metabolites are a rich source of biologically active compounds that can be used to develop new chemicals for pharmaceuticals. Plants contain a diverse group of highly valuable and accessible secondary metabolite resources such as tannins, terpenoids, alkaloids, and flavonoids, which have been found to have important pharmacological properties [3]. In general, essential oils and extracts of many plant species are considered nonphytotoxic compounds and are being studied for their antimicrobial, anti-inflammatory, antioxidant, antimutagenic, and preventive effects against cancer [4].

Medicinal plants and plant extracts with a complex of biologically active substances can be promising raw materials for producing antimicrobial components [2]. A certain ratio of secondary metabolites, which ensures beneficial effect on a living organism and pharmacological effect (antimicrobial, antioxidant, prebiotic properties), is a salient feature of plant extracts. It should be noted that the biological activity depends on the qualitative and quantitative composition of the secondary metabolites of the medicinal plant, which is not constant [5]. The content of secondary metabolites depends on the climatic conditions of the region, the chemical composition of the soil in which the medicinal plant grows, as well as the ecological situation in the area of cultivation [6]. The Baltic states with a temperate climate and small anthropogenic impact have the potential to grow plants with unique characteristics of both qualitative and quantitative content of biologically active components [7].

The chemical and spatial structures of secondary metabolites of medicinal plants are comparable to the metabolic products of microorganisms, which contributes to the fact that plant extracts of a complex of biologically active substances (BAS) are more actively involved in biological processes [8]. It is promising to study the biological activity of plant extracts of the BAS complexes isolated from medicinal plants growing in the Baltic states: *Cotinus coggygria*, *Lunaria rediviva*, *Sarothamnus scoparius*, *Eryngium maritimum*, *Goodyera repens*, *Corallorhiza trifida*, *Listera cordata*, *Dactylorhiza inaculata*, *Platanthera chlorantha*, *P. bifolia*.

The *Cotinus coggygria* biomass is used as a medicinal raw material. The medicinal raw material is harvested during the period from June to August [9]. The most valuable secondary metabolites found in *Cotinus coggygria* are tannins, the content of which varies from 6 to 30%, depending on the time of harvest and the amount of sun absorbed by the leaf [10]. Secondary metabolites have astringent, anti-inflammatory, and antiseptic properties [11]. Other parts of *Cotinus coggygria* are also interesting; for example, a root-based decoction has antipyretic properties [12]. Water and alcohol extracts from bush wood have bactericidal properties [13]. Decoctions of various parts of *Cotinus coggygria* are used against stomatitis, pharyngitis. It is also worth noting that the *Cotinus coggygria* leaves are included in the pharmacopoeia [14].

*Cotinus coggygria* extracts had high antioxidant activity in reaction with DPPH (IC_50_ = 2.6 ± 0.4 and 3.8 ± 0.5 μg/mL, respectively, for the obtained samples) and could slow down or completely inhibit the peroxidation process of plant lipids [9]. Additionally, a plant extract from the *Cotinus coggygria* leaves and flowers had anticancer and antigenotoxic properties. Acetone, methanol, water, ethyl acetate, and hexane extracts of this plant showed antibacterial properties. An aqueous extract from the *Cotinus coggygria* leaves had hepatoprotective properties [15].

However, the quality and characteristics of plant raw materials depend on different indicators (geographic, climatic, agro-technological, etc.); therefore, it is essential to study medicinal plants from different growing areas [16]. This work aimed to study in vitro the properties and characteristics of extracts from *Cotinus coggygria,* growing in the Russian region of the Baltics.

For the first time, the optimal modes of producing *Cotinus coggygria* extracts in vitro were studied, the qualitative and quantitative BAS composition was determined, and the antimicrobial and cytotoxic activity of extract samples of *Cotinus coggygria* growing in the Russian region of the Baltics was studied.

## 2. Results

The optimal technological parameters of the production process of *Cotinus coggygria* extracts, enriched with BAS, were selected. The extraction temperature, duration, and hydromodule (the quantitative ratio of organic solvent and plant raw materials) were selected as the main technological parameters that affect the BAS extract yield [8].

Table 1 and Table 2 present results of optimisation of technological parameters of the production process of *Cotinus coggygria* extracts, enriched with BAS, using 70% ethanol as an extractant.

**Figure 1:**
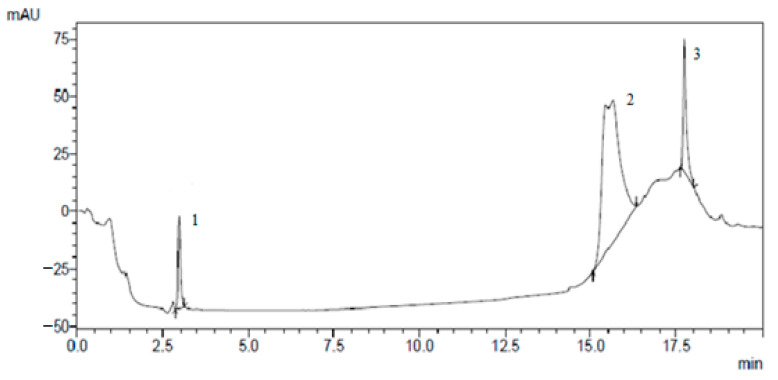
Preparative isolation of (1) rutin, (2) hyperoside, and (3) ferulic acid.

**Figure 2:**
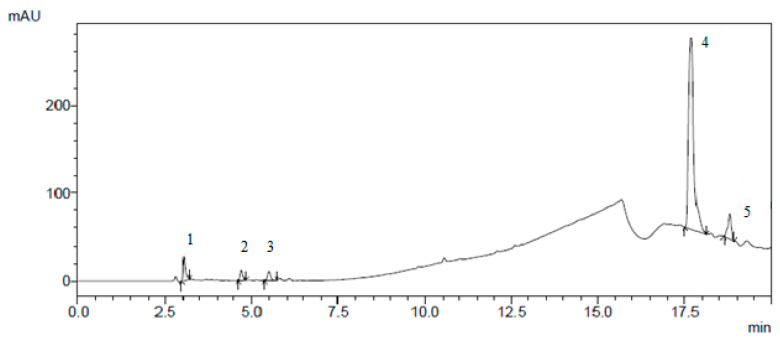
Preparative isolation of (1) disulphuretin, (2) sulphuretin, (3) sulphurein, (4) quercetin, and (5) kaempferol.

**Figure 3:**
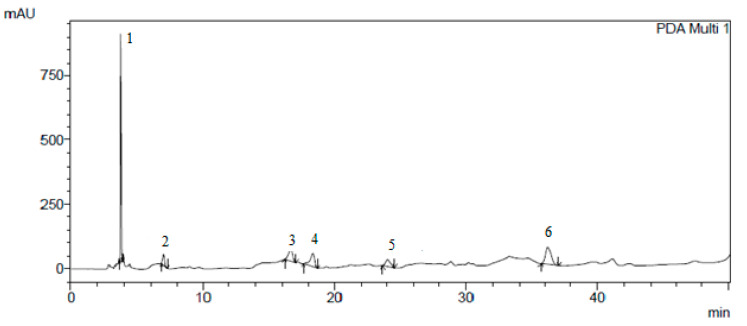
Hydroxyflavones isolated by preparative chromatography: 1—apigenin; 2—rutin; 3—hyperoside; 4—ferulic acid; 5—quercetin; 6—kaempferol.

**Figure 4:**
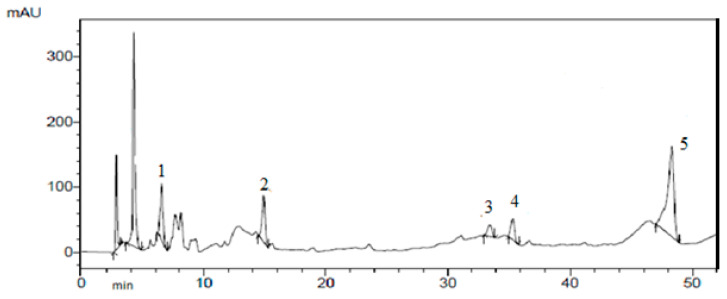
Derivatives of gallic and benzoic acids isolated by preparative chromatography: 1—7-*O*-β-D glucopyranoside; 2—pentagalloyl glucose; 3—methyl gallate; 4—3-*O*-α-L-rhamnofuranoside; 5—gallic acid.

The results of the isolation of individual biologically active substances from the *Cotinus coggygria* extracts by preparative chromatography are presented in Figure 1, Figure 2, Figure 3 and Figure 4.

In this work, we analysed the chemical composition of extracts obtained from the medicinal plant *Cotinus coggygria* growing in the Kaliningrad region. The qualitative composition of the extract samples from fresh or dried plant materials did not differ significantly. The qualitative and quantitative composition of biologically active substances of *Cotinus coggygria* extracts are presented in Table 3. The quantitative composition of BAS was determined by the area of the peaks obtained by HPLC.

The mercury content in the studied samples of plant BAS complex extracts did not exceed 0.0001 mg/kg. The cadmium and lead contents were within the normal range and was less than 0.002 mg/kg (for each indicator). It was experimentally established that the arsenic content in plant extracts did not exceed 0.08 mg/kg. The sum of α-, β-, and γ-isomers of HCH (hexachlorocyclohexane) in the studied samples did not exceed 0.001 mg/kg, and the content of 1,1,1-trichloro-2,2-bis(4-chlorophenyl) ethane (DDT) and its metabolites did not exceed 0.007 mg/kg. The content of aflatoxin B1 and radionuclides, plant extracts of the BAS complex, were within the normal range [8].

Strains causing diseases in humans and animals were chosen as test strains for the study of the antimicrobial activity of *Cotinus coggygria* extracts. These microorganisms can be introduced to the animal’s diet in case of violation of feed storage regimes, as well as by an employee during the preparation of compound feed for farm animals. *E. coli* is an opportunistic bacterium that causes gastroenteritis in animals and humans. *Staphylococcus aureus* is a pathogenic bacterium that causes toxic shock and sepsis in animals and humans. *P. vulgaris* is an opportunistic bacterium that causes intestinal infections. *C. albicans* is a microscopic fungus that is the causative agent of opportunistic infections. *L. mesenteroides* is an opportunistic bacterium that causes infectious diseases in animals and humans. Table 4 presents the results of evaluating the antimicrobial activity of the studied *Cotinus coggygria* extracts.

The results of studying the cytotoxicity of the *Cotinus coggygria* extract samples are presented in Table 5.

The influence of *Cotinus coggygria* extract samples on the activity of antioxidant enzymes is presented in Table 6.

When determining the anti-inflammatory activity of *Cotinus coggygria* extracts in vitro, it was found that the inhibition of COX-1 and COX-2 cyclooxygenase was 53.25% and 73.38%, respectively.

## 3. Discussion

Statistically significant differences (*p* > 0.05) in the extract yield (Table 1 and Table 2) with an extraction duration of up to 360 min were found at a hydromodule ratio of 1:5 in comparison with samples obtained at other hydromodule ratios. There were no statistically significant differences when changing the hydromodule from 1:1 to 1:20 with a duration of at least 120 min. At a hydromodule value of 1:5, no statistically significant differences were found with an extraction duration of 60 min or more. With an extraction duration of up to 360 min, the extract yield differed statistically significantly (*p* = 0.887–0.981) at a temperature of 60 °C, in comparison with the rest of the samples. At an extraction temperature of 40 °C and boiling, no statistically significant difference (*p* < 0.05) in the extract yield was found at an extraction duration of 60 to 180 min. In all other cases, the values of the extract yield significantly (*p* = 0.876–0.973) depended on such values as the extraction duration, hydromodule ratio, and temperature. The statistical evaluation of the effects of variable parameters on the extraction process optimisation confirmed their significance [17]. In the analysis of variance of the adequacy of the constructed model based on the results of the full factorial experiment, the corresponding confidence coefficients did not exceed 0.05 (*p* = 0.027 to 0.043). A combination of the theoretical and experimental research results shows that the maximum yield of BAS complex extract from *Cotinus coggygria* (Table 1 and Table 2) is achieved when the ratio of the solvent volume to the raw material weight is 1:5, the extraction process duration is 60 min, and the process temperature is 60 °C. Dry roots and stems of *Cotinus coggygria* were ground to powders, and the powders were extracted with 95% ethanol by liquid–liquid extraction [7]. Then, 100 g of ground powders of roots and stems were extracted with 95% ethanol (2000 mL) for 3 h.

The results obtained (Table 3) indicate that the extracts of *Cotinus coggygria* are enriched with biologically active substances prevailing in plant raw materials. In the study [8], the methanol extract of the *Cotinus coggygria* stem contained 3.78 mg of gallic acid per gram of dry plant material in total phenolic composition, while the content of flavonoids was 8.29 mg of rutin per gram of dry plant material. Myricetin was the main component of the extract (511.5 μg/g). In the extract [8], hydroxyl derivatives of cinnamic acids (chlorogenic, caffeic, coumaric, ferulic, and rosmarinic acids) were identified in various amounts. Rosmarinic acid (18.55 μg/g) was the main phenolic acid in the extract, while other phenolic acids were present in lesser amounts. Our data on the BAS content diversity are in good agreement with the results carried out by Matic et al. [8].

The study by Wang et al. [7] asserted that *Cotinus coggygria* extracts contain sulphuretin, fisetin, dastin, quercetin, taxifolin, gallic acid, 3′,4′,7-trihydroxyflavanone, disulphuretin, and myricetin. The highest content of total phenolic compounds (92.9%), tannins (83.4%), and flavonoids (3.5%) was determined in the ethyl acetate fraction from crushed and dried young shoots, compared to chloroform and aqueous fraction. In this fraction, gallic acid, apigenin, luteolin, and their derivatives were detected by HPLC. The acetone extract of *Cotinus coggygria* young shoots is characterised by the presence of gallic acid, gallic acid derivatives, and flavonol kaempferol-3-*O*-glucoside. The ethyl acetate fraction contained gallic acid, gallic acid derivatives, kaempferol-3-*O*-glucoside, and flavones luteolin-7-*O*-glucoside, luteolin-8C-glucoside (orientin), apigenin glycoside, and apigenin [7].

It is known that the toxicological indicators of plant raw materials and BAS complex extracts from medicinal plants are not constant and depend on the composition of the soil and the ecological situation of the growing region. Microbiological indicators of the feedstock intended for the production of feed additives [9,10] for farm animals are also not constant and depend on the sanitary and hygienic state of production facilities. In this regard, the toxicological and microbiological indicators of the safety of plant extracts should be regularly studied. Empirical data obtained in the course of studying the quantitative content of heavy metals in BAS complex extracts from *Cotinus coggygria* growing in the Russian region of the Baltics show that the resulting extracts have a low content of such metals as mercury, cadmium, lead, arsenic; all toxicological indicators are within the normal range. The study of toxicological indicators of other territories shows that the content of toxic elements, such as cadmium and lead, in samples of medicinal plants growing in the neighbouring territories of Dubna, Moscow region, does not exceed the threshold limit value for heavy metals proposed by the State Pharmacopoeia of the Russian Federation. At the same time, attention is drawn to the fact that in most of the studied plants, the content of lead, cadmium, copper, and zinc exceeds the maximum allowable concentration for dry vegetables and fruits [11].

The studied *Cotinus coggygria* extracts (Table 4) have antimicrobial properties against the tested strains (*E. coli, S. aureus, P. vulgaris, C. albicans, L. mesenteroides*). The evidence of the antimicrobial activity of medicinal plants was presented by Marčetić et al. [12]. This study presented the comparative chemical composition and antimicrobial activity of citronella (*Cymbopogon* spp.). Extracts, produced by various methods, namely, hydrodistillation and steam distillation, were determined using gel chromatography, disk diffusion analysis, and analysis in microdilution broth. The main components of the extract were citronellal (2.2–55.4%), geraniol (14.2–53.0%), citronellol (8.2–16.4%), isopulegol (0.3–12.6%), elemol (0.8–8.2%), and limonene (0.2–5.0%). Shameem et al. [18] studied the antimicrobial activity of young shoots of *Cotinus coggygria*. Acetone extract and a derivative ethyl acetate fraction effectively suppressed the growth of Gram-positive and Gram-negative bacteria (MIC 25–200 μg/mL), while the chloroform fraction exhibited pronounced activity against the *Candida albicans* yeast (MIC 3.12 μg/mL).

The antioxidant activity of *Cotinus coggygria* extracts was 145.09 ± 7.25 mg AA/g, which indicates the high antioxidant characteristics of the obtained extracts [8]. Our results are comparable with those presented in [18]. The ethyl acetate fraction exhibited a significant ability to reduce iron (10.7 mmol Fe 2+/g of extract) and very high activity in trapping DPPH radicals (IC_50_ = 1.7 μg/mL) and inhibition of lipid peroxidation (IC_50_ = 41.8 μg/mL). High amounts of total phenols (929.8 mg/g), tannins (833.8 mg/g), and flavonoids (35.5 mg/g) were detected in the ethyl acetate fraction, which also had significant anti-inflammatory (76.7%) and cytotoxic effects (IC_50_ = 15.6 μg/mL).

As a result of evaluating the effect of *Cotinus coggygria* extract samples on the activity of antioxidant enzymes (superoxide dismutase, glutathione peroxidase, and catalase), it was found that the level of the reduced form of glutathione under the action of antioxidant enzymes is significantly higher than that of the oxidised form, which indicates a significant decrease in free radical processes [9].

The article [18] presented in vitro experiments confirming wound-healing, anti-inflammatory, antibacterial, cytotoxic, antioxidant, hepatoprotective, and antidiabetic effects. The metabolites of *Cotinus coggygria* responsible for the main pharmacological effects of this plant were noted. The review compared *Cotinus coggygria* and *Toxicodendron vernicifluum*. The comparative approach was aimed at opening new perspectives in the study of *Cotinus coggygria*, suggesting optimal use in therapy. The relevance of the chemosystematic approach in the study of medicinal plants was noted.

The cytotoxicity and antioxidant properties of methanol extracts of *Cotinus coggygria* leaves and flowers, as well as their chemical composition, were studied in [19]. Extracts of *C. coggygria* flowers and leaves showed better antioxidant activity in the reaction with the 1,1-diphenyl-2-picrylhydrazyl (DPPH) radical and in inhibiting lipid peroxidation (LP) than *C. mas* extracts. Preliminary results showed that all extracts have potential cytotoxic activity against HeLa and LS174 human cancer cell lines in vitro with a stronger inhibition of HeLa cell growth than LS174 cell growth. The cytotoxic activity of *C. coggygria* extract samples showed a significant correlation with their in vitro antioxidant activity. Brine shrimp lethality bioassay did not show significant changes in toxicity.

In [20], Guava fruit extracts were analysed for antioxidant activity measured in methanol extract (AOAM), antioxidant activity measured in dichloromethane extract (AOAD), ascorbic acid, total phenolics, and total carotenoids contents. The ABTS, DPPH, and FRAP assays were used for determining both AOAM and AOAD, whereas the ORAC was used for determining only AOAM. Averaged AOAM were 31.1, 25.2, 26.1, and 21.3 mM Trolox equivalent/g fresh mass (mM TE/g FM), as determined by the ABTS, DPPH, FRAP, and ORAC assays, respectively. Averaged AOAD were 0.44, 0.27, and 0.16 mM TE/g FM, as determined by the ABTS, DPPH, and FRAP assays, respectively. AOAM determined by all assays were well correlated with ascorbic acid (0.61 ≤ r ≤ 0.92) and total phenolics (0.81 ≤ r ≤ 0.97) and also among themselves (0.68 ≤ r ≤ 0.97) but had a negative correlation with total carotenoids (−0.67 ≤ r ≤ −0.81).

The COX-1 inhibition under the action of *Cissus quadran-gularis, Plumbago zeylanica, Terminalia bellarica,* and *Terminalia chebulla* extracts ranged from 25.91% to 61.83% [21], which correlates well with the results of our studies.

## 4. Materials and Methods

### 4.1. Research Objects

Extracts of *Cotinus coggygria* (*Cotinus coggygria* Scop., family *Anacardiaceae*) were the research objects. The medicinal plant growing in the Russian region of the Baltics was the raw material for the production of extracts. The biomaterial of the species was confirmed by A.V. Pungin, head of the herbarium at the Institute of Living Systems, IKBFU. Aerial parts of mature plants were harvested to produce extracts [18]. In the collected biomass, the ratio of shoots/leaves/flowers of each plant averaged 4:2:1.

### 4.2. Selection of Extraction Parameters

The dependence of the *Cotinus coggygria* extract yield on the hydromodule and temperature at different durations of the process was studied. The hydromodule was changed in the ratio of 1:1, 1:2, 1:5, 1:10, and 1:20. The duration of the extraction process varied from 10 to 360 min. The temperature varied from 25 to boiling. Conclusions regarding the optimal regimes were drawn based on the highest extract yield. The extract yield was calculated as % of the total biomass of the medicinal plant.

When selecting the optimal technological parameters for the process of obtaining *Cotinus coggygria* extracts, the extraction of biologically active substances was carried out as follows: a portion of the test sample was weighed on an analytical balance (Oxaus PX85, New York, NY, USA), transferred into a Falcon polyethylene test tube, an organic solvent (ethanol) was poured in the amount of 1:5 according to the experiment procedure, and extraction process carried out. The duration and temperature varied up to 360 min and from 25 °C to boiling, respectively. Further, the filtration process was carried out, followed by centrifugation of the filtrate at a rotor speed of 3900 ± 100 rpm. The filtrate was centrifuged in a PE-6900 centrifuge (Ekros, Moscow, Russia) to remove suspended particles. The solvent was evaporated from the extract on an IKA RV 8 V rotary evaporator (IKA, Staufen, Germany) under reduced pressure from a pre-weighed on a CAS CUW420H balance (CAS Corporation Ltd., Seoul, Korea) 100 mL flask. The flask was weighed and the yield of the extract was determined [6].

### 4.3. Selection of Parameters for Individual BAS Extraction from the Extract

Individual BAS samples were obtained by separating the *Cotinus coggygria* extract on a glass chromatographic column by preparative HPLC using a Shimadzu LC-20AD chromatograph (Shimadzu, Kyoto, Japan) and a high-pressure steel column with a sorbent size of 2.5 μm, a diameter of 2.5 mm, and a length of 250 mm [22]. Elution conditions: flow rate 1 mL/min; eluent water: methanol: 0.1% trifluoroacetic acid with a linear gradient from 40% to 90% methanol in 20 min; detection at a wavelength of 254 nm.

The benzoic acid and gallic acid derivatives were isolated from *Cotinus coggygria* by HPLC using a column with a sorbent modified with a nitrile phase, sorbent particle size 1.8 μm, diameter 2.5 mm, length 250 mm. Eluent—water: acetonitrile, 0.1% trimethylamine; linear gradient from 15% to 85% acetonitrile; elution time 55 min. Individual substances were collected automatically using a fraction collector [19].

### 4.4. Study of the Chemical Composition of Plant Extracts

To study the chemical composition of plant extracts [23], we used the HPLC method (Chromatograph Shimadzu LC-20AD, Kyoto, Japan) according to GPM.1.2.1.2.0005.15; the detection was carried out using a diode array detector in the detection range of 180–900 nm, and the flow rate of the eluent in all cases was 1 mL/min. Elution was carried out in a gradient mode, and the time and gradient were selected individually for each separation case; a mixture of prepared water (purification level MQ) and acetonitrile with the addition of 0.1% trifluoroacetic acid was used as solvents; separation was carried out on a Phenomenex reversed-phase column (Phenomenex, Ashburn, VA, USA) with sorbent silica gel (column size: 250 mm diameter, 2.5 mm length; particle size: 25 μm) modified with C-18, with phenyl endcapping [24].

### 4.5. Analytical Standards

Isorhamnetin 3-glucopyranoside (Isorhamnetin-3-*O*-β-D glucopyranosyl-(1→2)-β-D glucopyranosyl-(1→6)-β-D-glucopyranoside, ≥90%, 00960590), Kaempferol 3-glucoside (3,4′,5,7-Tetrahydroxyflavone 3-glucoside, 3-(β-D-Glucopyranosyloxy)-5,7-dihydroxy-2-(4-methoxyphenyl)-4H-1-benzopyran-4-one, 3-Glucosylkaempferol, Astragalin, Kaempferol 3-β-D-glucopyranoside, Kaempferol 3-glucoside, ≥90%, 68437), Apigenin 7-glucoside (4′,5,7-Trihydroxyflavone 7-glucoside, ≥97%, 44692), Chlorogenic acid (1,4,5-Trihydroxycyclohexanecarboxylic acid 3-(3,4-dihydroxycinnamate), ≥95%, C3878), Ruthin (3,3′,4′,5,7-Pentahydroxyflavone 3-rutinoside, 3-Rutinosylquercetin, Globulariacitrin, Ilixanthi, Myrticalorin, Osyritrin, Paliuroside, Phytomelin, Quercetin 3-rhamnoglucoside, Quercetin 3-rutinoside, Rutoside, Sophorin, Tanrutin, Violaquercitrin, ≥95.0%, PHL89270), Hyperoside (3,3′,4′,5,7-Pentahydroxyflavone 3-D-galactoside, Hyperin, Quercetin 3-D-galactoside, primary reference standard, 00180585), Ferulic acid (trans-4-Hydroxy-3-methoxycinnamic acid, trans-Ferulic acid, 99%, 128708), Quercetin (2-(3,4-Dihydroxyphenyl)-3,5,7-trihydroxy-4H-1-benzopyran-4-one, 3,3′,4′,5,6-Pentahydroxyflavone, ≥95.0%, Q4951), Kaempferol (3,4′,5,7-Tetrahydroxyflavone, 3,5,7-Trihydroxy-2-(4-hydroxyphenyl)-4*H*-1-benzopyran-4-one, Robigenin, ≥97.0%, 60010), Disulphuretin (certified reference material, CAS: 97-77-8), Sulphuretin (sulphurin, Sulphuretin, 2-(3,4-Dihydroxybenzylidene)-6-hydroxy-3(2H)-benzofuranone, 7,3′,4′-trihydroxyaurone, 3′,4′,6-trihydroxyaurone, certified reference material, CAS: 120-05-8), Gallic acid (3,4,5-Trihydroxybenzoic acid, ≥97.5%, G7384), Methyl gallate (Gallic acid methyl ester, Methyl 3,4,5-trihydroxybenzoate, ≥90.0%, PHL82592), Pentagalloyl glucose (certified reference material, CAS: 50678-27-8), 3,3′,4′,5,6,7–hexahydroxyflavonone (certified reference material, CAS: 27200-12-0), 3,3′,4′,5,5′,7– hexahydroxyflavonone (Dihydromyricetin, (2R,3R)-3,5,7-Trihydroxy-2-(3,4,5-trihydroxyphenyl)-2,3-dihydrochromen-4-one, Ampelopsin, Ampeloptin, DHM, ≥95.0%, 42866), 3-*O*-α-L-rhamnopyranoside (certified reference material, CAS: 1381853-30-0), 3,3′,4′,5,5′,7–hexahydroxyflavulium(1+) (certified reference material, CAS: 528-53-0), 7-*O*-β-D glucopyranoside (certified reference material, CAS: 4860-85-9) were purchased from Fluka/Sigma-Aldrich (Sigma-Aldrich Rus, Moscow, Russia).

For the chromatographic determination of substances, a mixed initial solution was prepared immediately before the experiment, containing 1 mg/mL of each of the substances under study in ethanol. A set of standard solutions for the calibration curve were prepared by sequential dilution of the mixed stock solution with ethanol to final concentrations from 0.1 to 100 μg/mL. The solutions were chromatographed and eluted as described above. The content of each of the studied substances in the extracts was calculated based on the specified calibration curves between the peak regions and the concentrations of the standard solutions.

### 4.6. Determination of the Heavy Metal Content in Plant Extracts

The quantitative content of mercury in the obtained extracts was determined by a colourimetric method based on the destruction of plant raw materials.

The retention of cadmium salts in plant extracts was determined by the method of polarisation in the alternating current mode.

The polarogram was recorded at a voltage from −0.4 to −0.8 V relative to the bottom mercury, choosing the operating mode in accordance with the instructions for the polarograph.

The arsenic content was determined by the method described in GOST 26930-86. The ash obtained by dry mineralisation was carefully dissolved in a 30–50 cm^3^ solution of hydrochloric acid with a concentration (HCl) = 0.3 mol/dm^3^ and, avoiding splashing, hydrochloric acid with a density of 1.19 g/cm^3^ was added at the rate of 4 cm^3^ acid per 1 g of magnesium oxide added in the sample before ashing. If the ash did not dissolve well, it was heated with hydrochloric acid in a water bath. The resulting ash solution was used for subsequent testing. According to the obtained value of optical density, the mass of arsenic was found using a calibration graph.

### 4.7. Study of Toxicological Indicators of Plant Extracts

The following analytical and laboratory equipment was used to study the toxicological safety indicators of *Cotinus coggygria* extracts: an AAS Solaar MK II atomic absorption spectrometer (Agelent, Melbourne, Australia), a photoelectric colourimeter KFK-3 (ZOMZ, Sergiev Posad, Russia). The content of pesticides, aflatoxin B1, and radionuclides in the obtained *Cotinus coggygria* extracts was determined using a beta spectrometer, devices (radiometer, spectrometer) for measuring the activity of radionuclides in a load [8].

The optical density was measured on a vertical photometer with a measurement range of optical density from 0 to 2.5, with a maximum permissible absolute single measurement error of no more than ±0.005, supplied with an interference filter for wavelengths from 490 to 492 nm. Aflatoxin B_1_ content *X_B_*_1_ in each parallel sample, mg/kg, was calculated by the formula

*X*_*B*1_ = *C·V·K*/*m*,

where *C*—mass concentration of aflatoxin *B_1_* in the working solution of the extract, μg/cm^3^; m—weighed sample taken for analysis, g; *V*—volume of the acetonitrile and water mixture taken for extraction, cm^3^; *K*—the multiplicity of dilution of the extract when preparing the working solution.

The method for determining the number of mesophilic aerobic and facultative anaerobic microorganisms by inoculating the agar nutrient media is based on inoculation of the product or diluting a sample of the product into a nutrient medium, incubating the culture, and counting all visible colonies that have grown [25].

The method for the determination of toxin-forming anaerobes is based on inoculating a certain amount of the extract or its dilutions into a liquid selective medium, cultivating at (36 ± 1) °C for 48 h, then reinoculating the grown cultures on dense differential diagnostic media and cultivating at (36 ± 1) °C for 48 h, isolating characteristic colonies, and finally, confirmation by biochemical tests of their belonging to bacteria of the genera *Proteus* and(or) *Morganella* and(or) *Providencia*, or to the species *Proteus vulgaris* or *Proteus mirabilis*. If the found microorganisms deaminated phenylalanine, they were identified as bacteria of the genera *Proteus, Morganella, Providencia*.

The method for detecting pathogenic microorganisms is based on the detection of nucleic acid sequences (DNA or RNA) by molecular methods based on polymerase chain reaction (PCR). Different sequences of target microorganisms or viruses were determined with efficient amplification, even if differences in primer sequences and sample binding sites were reported as indicated in the field of application of the method. At least 50 strains of target microorganisms or viruses were analysed. Pathogens that required qualitative testing were detected at levels of 1 cell in 10 cells.

### 4.8. Determination of the Antimicrobial and Antifungal Activity of Plant Extracts

The in vitro antimicrobial activity of plant extracts was determined against the growth of opportunistic and pathogenic test strains of microorganisms by two methods: the diffusion method (on a solid nutrient medium) and a method based on measuring the optical density (in a liquid nutrient medium) [26].

As test cultures, we used strains *E. coli* ATCC 25922, *S. aureus* ATCC 25923, *P. vulgaris* ATCC 63, *C. albicans* EMTK 34, *L. mesenteroides* EMTK 1865, *Penicillium expansum* purchased from the State Collection of Pathogenic Microorganisms and Cell Cultures (GKPM-Obolensk, Obolensk, Russia). The following equipment was used: a laminar flow cabinet (class 2) A BAVp-01-“Laminar-S”-1.5 (Laminarniye systemy, Miass, Russia), a shaking incubator LSI-3016A (Daihan Labtech Co., Ltd., Seoul, South Korea), an autoclave DGM-80 (Pharma Apparate Handel AG, Zug, Switzerland), upright microscope AxioScope A1 (Zeiss, Jena, Germany).

*E. coli* strain was cultured on a solid LB nutrient medium and a liquid LB nutrient medium at a temperature of 37 °C. *S. aureus* strain was cultured on milk salt agar using feed yeast hydrolysate in an amount of 24.0 g, sodium chloride (NaCl)—75.0 g. The cultivation process was carried out at a temperature of 36 °C. The opportunistic bacterium *P. vulgaris* was cultured in meat peptone broth at a temperature of 36 °C. The microscopic fungus *C. albicans* was cultured out in Sabouraud nutrient medium at a temperature of 25 °C. The *L. mesenteroides* strain was cultivated on a solid nutrient medium (yeast extract 4.0%, meat extract 10.0%, casein hydrolysate 10.0% glucose 20.0%, ammonium citrate 2.0% sodium acetate 5.0%, Tween 80 1.0%, disubstituted potassium phosphate 2.0%, sulphate magnesium 0.2%, manganese sulphate 0.05%) at a temperature of 36 °C. All standard media were purchased from Khimreaktivsnab, Ufa, Russia.

In the diffusion method for determining the antimicrobial activity of the extracts, the test strain was inoculated on an agar nutrient medium using a bacterial lawn technique, and at the same time, the BAS complex extracts were placed on the lawn. A paper disc with a nutrient medium was used as a control, and a disc with antibiotic rifampicin (from a standard kit) was used as a reference drug. Petri dishes were incubated at a temperature corresponding to the optimal growth temperature of each test strain of the microorganism for 24.0 ± 0.5 h. The results were determined by the presence and size (in mm) of the transparent zone of the absence of microorganism growth around the disc [27].

In the case of using a method based on measuring optical density (in a liquid nutrient medium), to assess the antimicrobial effect of plant extracts, cell cultures were co-incubated with the studied extracts in 96-well culture plates. Overnight broth cultures were resuspended in Mueller–Hinton medium (*C. albicans*—in Sabouraud medium), bringing the number of microorganisms to an inoculation dose of ~10^5^ CFU/mL. Cell suspension and the studied extracts were simultaneously introduced into the wells in an amount of 1/10 of the total volume; control—MRS; reference drug—rifampicin (10 μg/mL). The total volume of the suspension in the well is 200 μL; the number of repetitions—2; incubated at 36 ± 1 °C on a shaker (580 rpm). After 24.0 ± 0.5 h, the optical density (OD) was measured using a multi-reader at a wavelength of 595 nm. The presence of bactericidal activity was assessed by the change in OD in comparison with the control. OD was lower in wells where cell growth stopped or slowed down than in wells with normal growth of microorganisms.

### 4.9. Determination of the Cytotoxicity of Extracts

The cytotoxicity of the dried biomass extracts was studied in vitro on human glioblastoma cells, Hep-G2, MCF-7, A549, HCT116 in an intermediate exposure mode (24 h). The colourimetric test was based on the reduction of the yellow salt of MMT (3-(4,5-dimethylthiazol-2-yl)-2,5-diphenyltetrazole bromide) to purple formazan crystals by metabolically active cells. Insoluble formazan was transferred into solution with a special buffer, which allows determining the intensity of the staining. The colour intensity of the solution is proportional to the number of living metabolically active cells. The cytotoxic effect of the extract was at IC_50_ level, and the concentration range of the extract used was 500–2500 μg/mL.

The measurements were performed on a ClarioSTAR microplate reader at a wavelength of 590 nm. The cells (1 × 10^4^) were seeded into a 96-well flat-bottomed plate. MTT without cells was used as a control. All experiments were carried out in triplicates. The cells were incubated for 24 h at 37 °C in a 5% CO_2_ medium. Then, the extracts were added and incubated (short exposure 1 h, intermediate exposure 24 h, long exposure 48–72 h).

After the incubation period, 10 μL of MTT reagent (final concentration 0.5 mg/mL) was added to each well. The plate was incubated for another 3 h (37 °C, 5.0–6.5% CO_2_). Then, the culture medium was removed, and 100 μL of MTT solvent was added to each well. The plate was covered with foil and incubated in an orbital shaker for 15 min. The colour intensity was determined at 590 nm. The reference filter was set at 620 nm.

### 4.10. Determination of the Antioxidant Activity of Plant Extracts

The antioxidant activity of plant extracts was determined by their ability to reduce the 2,2-diphenyl-1-picrylhydrazyl radical (DPPH, C_18_H_12_N_5_O_6_, M = 394.33). The reaction of the interaction of antioxidants with DPPH-radical proceeds according to the scheme:

DPPH* + AH → DPPH–H + A*

When the DPPH-radical is reduced by an antioxidant, the purple-blue colour of DPPH in ethanol decreases, and the reaction is monitored by the change in optical density using conventional spectrophotometric methods [28].

For the analysis, plant extracts were mixed with 2.85 mL of a freshly prepared 0.1 mM solution of 2,2-diphenyl-1-picrylhydrazyl. The mixture was incubated in the dark at room temperature for 30 min. The decrease in optical density, compared to the control (70% methanol solution), was recorded at 517 nm (UV-3600 spectrophotometer, Shimadzu, Kyoto, Japan). As standard solutions, we used solutions of ascorbic acid (AA) of known concentration. The results of the analyses were expressed in mg AA equivalent per gram of dry weight of plant extracts (mg AA/g). The antioxidant activity of the samples was analysed in triplicate [29].

All chemicals (analytical or higher grade) used in this study were purchased from Fluka/Sigma-Aldrich (Sigma-Aldrich Rus, Moscow, Russia).

### 4.11. Study of the Effect of Extracts on the Activity of Antioxidant Enzymes

The effect of *Cotinus coggygria* extracts on the activity of antioxidant enzymes was confirmed by fluorescence methods using antioxidant markers 2,2-azinobis (3ehtylbenzothiazoline-6-sulfonic acid) diammonium salt (ABTS), the oxygen radical absorbance capacity (ORAC), Ferric reducing antioxidant power (FRAP), diphenyl-1-picrylhydrazyl (DPPH), superoxide dismutase (SOD), 6-hydroxy-2,5,7,8-tetramethylchroman-2-carboxylic acid (Trolox)-equivalent antioxidant capacity (TEAC).

### 4.12. Study of the Anti-Inflammatory Activity of Extracts

The anti-inflammatory activity was studied in vitro. The effect of *Cotinus coggygria* extract samples on COX-1 and COX-2 cyclooxygenase was determined by measuring prostaglandin E2 (PGE2) using a gamma COX (ovine/human) inhibitor screening assay kit for screening COX inhibitors (Cayman Chemicals, USA). The inhibitory activity of the tested compounds was evaluated according to the following formula [30]:

$$COX_{inhibition\;activity}=1-\frac{T}{C}\times 100\%,$$

where *T*—absorption in the presence of an inhibitor; *C*—absorption at 100% of the initial enzyme activity without inhibitor.

### 4.13. Statistical Analysis

Each experiment was repeated three times, and the data are expressed as means ± standard deviation. Post hoc analysis (Tukey test) was undertaken to identify samples that were significantly different from each other. The equality of the variances of the extracted samples was checked using the Levene test. A factorial experiment (3^2^ factorial design) with variable parameters (factors) of the hydromodule, temperature, and extraction duration was conducted to determine the optimal extraction modes. Three levels were considered for each factor: 1:1, 1:5, 1:20 for hydromodule; 25 °C, 45 °C, boiling for temperature; 0, 180, and 360 min for duration. The extract yield (%) was considered as a target function. The adequacy of the constructed model was verified using an analysis of variance (ANOVA). The data were subjected to ANOVA using Statistica 10.0 (StatSoft Inc., 2007, Tulsa, OK, USA). Differences between means were considered significant when the confidence interval was below 5% (*p* < 0.05).

## 5. Conclusions

The influence of the extraction parameters (hydromodule, temperature, and extraction time) on the qualitative and quantitative composition of BAS of the *Cotinus coggygria* extract samples and their toxicological and cytotoxic properties were studied. The antioxidant and antimicrobial properties, as well as composition of biologically active substances of *Cotinus coggygria* growing in the Russian region of the Baltics, were studied in vitro for the first time. As the result of these studies, the chemical composition, the BAS content, the content of heavy metals, toxicological properties, and in vitro antimicrobial and antioxidant activity of the medicinal plant *Cotinus coggygria* were screened for the first time. Among this medicinal plant BAS, flavonoids have been identified that are of interest for the production of feed additives for livestock as an alternative to antibiotics.

Plant-based feed additives are now widely used for the treatment and prevention of many diseases of livestock and poultry. Their range is expanding annually and the number of phytopreparations is increasing. The advantages of phytopreparations for livestock and poultry over synthetic drugs are their mild action and low toxicity.

The use of medicinal plants for the production of feed additives is relevant in terms of improving regional economies, as well as improving the quality of life and nation’s health by providing ecologically clean livestock products.

## Figures and Tables

**Table 1:** The *Cotinus coggygria* extract yield (%) at various hydromodule indicators and extraction process duration.

Hydromodule	Process Duration, min					
	10	30	60	120	180	360
1:1	2.52 ± 0.07 ^a/a^	3.81 ± 0.11 ^a/a^	5.22 ± 0.15 ^a/b^	7.29 ± 0.22 ^a/c^	7.38 ± 0.22 ^a/c^	7.38 ± 0.22 ^a/c^
1:2	2.88 ± 0.08 ^a/a^	3.94 ± 0.11 ^a/ab^	4.35 ± 0.13 ^a/b^	6.58 ± 0.20 ^a/c^	7.67 ± 0.23 ^a/c^	7.71 ± 0.24 ^a/c^
1:5	6.24 ± 0.18 ^b/a^	8.83 ± 0.26 ^b/b^	8.78 ± 0.26 ^b/b^	8.88 ± 0.26 ^b/b^	9.95 ± 0.29 ^b/b^	8.81 ± 0.25 ^b/b^
1:10	3.48 ± 0.10 ^a/a^	3.98 ± 0.12 ^a/a^	2.98 ± 0.09 ^c/a^	5.94 ± 0.18 ^a/b^	5.97 ± 0.18 ^a/b^	6.04 ± 0.19 ^a/b^
1:20	2.42 ± 0.07 ^a/a^	3.01 ± 0.09 ^a/a^	5.01 ± 0.15 ^a/b^	5.95 ± 0.18 ^a/bc^	6.01 ± 0.18 ^a/c^	6.07 ± 0.18 ^a/c^

Data presented as a mean ± SD (*n* = 3). Values in columns/rows followed by the same letter do not differ significantly (*p* > 0.05), as assessed by post hoc test (Tukey test).

**Table 2:** The *Cotinus coggygria* extract yield (%) at various temperature indicators and extraction process duration.

Temperature, °C	Process Duration, min					
	10	30	60	120	180	360
25	6.21 ± 0.18 ^a/a^	8.83 ± 0.26 ^a/b^	8.78 ± 0.26 ^a/b^	8.88 ± 0.26 ^a/b^	9.95 ± 0.29 ^a/c^	8.81 ± 0.26 ^a/b^
40	7.55 ± 0.22 ^b/a^	7.98 ± 0.24 ^a/a^	8.92 ± 0.27 ^a/b^	9.21 ± 0.27 ^a/b^	8.18 ± 0.24 ^b/ab^	7.24 ± 0.22 ^b/a^
60	9.79 ± 0.29 ^c/a^	12.35 ± 0.37 ^b/b^	16.98 ± 0.51 ^b/c^	14.05 ± 0.42 ^b/d^	14.01 ± 0.42 ^c/d^	13.12 ± 0.39 ^c/e^
Boiling	7.62 ± 0.23 ^b/a^	8.14 ± 0.24 ^a/a^	14.04 ± 0.42 ^c/b^	14.12 ± 0.42 ^b/b^	14.14 ± 0.42 ^c/b^	12.17 ± 0.36 ^d/c^

Data presented as a mean ± SD (*n* = 3). Values in columns/rows followed by the same letter do not differ significantly (*p* > 0.05), as assessed by post hoc test (Tukey test).

**Table 3:** The quantitative composition of individual BAS in the *Cotinus coggygria* extract.

BAS	BAS Content, mg/kg
Rutin	51.88 ± 1.55
Hyperoside	40.67 ± 1.22
Ferulic acid	5.62 ± 0.16
Quercetin	15.04 ± 0.45
Kaempferol	14.33 ± 0.42
Disulphuretin	0.240 ± 0.007
Sulphuretin	1.11 ± 0.03
Sulphurein	0.270 ± 0.008
Gallic acid	4.81 ± 0.14
Methyl gallate	3.51 ± 0.10
Pentagalloyl glucose	21.14 ± 0.63
3,3′,4′,5,6,7–Hexahydroxyflavonone	15.92 ± 0.47
3,3′,4′,5,5′,7–Hexahydroxyflavonone	15.33 ± 0.46
3-*O*-α-L-rhamnofuranoside	8.72 ± 0.25
3,3′,4′,5,5′,7–Hexahydroxyflavulium(1+)	5.09 ± 0.15
7-*O*-β-D glucopyranoside	14.51 ± 0.43
3,3′,4′,7– Tetrahydroxyflavonone	7.77 ± 0.22

Data presented as a mean ± SD (*n* = 3).

**Table 4:** Indicators of antimicrobial activity (diameter of the lysis zone, mm) of the *Cotinus coggygria* extract.

Sample	Test Cultures					
	1	2	3	4	5	6
Extract	16.5 ± 0.5	14.5 ± 0.5	11.0 ± 0.5	13.0 ± 0.5	14.5 ± 0.5	13.0 ± 0.5
Control	0.0	0.0	0.0	0.0	0.0	0.0
Rifampicin	23.0 ± 0.5	21.0 ± 0.5	20.0 ± 0.5	19.5 ± 0.5	18.0 ± 0.5	18.0 ± 0.5

1—*E. coli*; 2—*S. aureus*; 3—*P. vulgaris*; 4—*C. albicans*; 5—*L. mesenteroides*; 6—*P. expansum*; control—test culture medium. Data presented as a mean ± SD (*n* = 3).

**Table 5:** Cytotoxicity indicators of medicinal plant *Cotinus coggygria* extract samples.

Sample	IC_50_, μg/mL				
	Glioblastoma Cells	Hep-G2	MCF-7	A549	HCT116
Extract	45.68 ± 2.26	65.47 ± 2.48	48.23 ± 2.30	32.40 ± 2.02	33.13 ± 2.03

Data presented as a mean ± SD (*n* = 3).

**Table 6:** The influence of *Cotinus coggygria* extract samples on the activity of antioxidant enzymes.

Sample	Antioxidant Markers, mM TE/g FM *					
	ABTS	ORAC	FRAP	DPPH	SOD	TEAC
Crocin (control)	0.13 ± 0.01 ^a^	0.16 ± 0.01 ^a^	0.10 ± 0.01 ^a^	0.13 ± 0.01 ^a^	0.14 ± 0.01 ^a^	0.16 ± 0.01 ^a^
Extract	0.46 ± 0.02 ^b^	0.21 ±0.01 ^a^	0.15 ±0.01 ^a^	0.29 ± 0.02 ^b^	0.35 ± 0.02 ^a^	0.26 ± 0.02 ^a^

* mM TE/g FM—mM Trolox equivalent/g fresh mass. Data presented as a mean ± SD (*n* = 3). Values in columns followed by the same letter do not differ significantly (*p* > 0.05), as assessed by post hoc test (Tukey test). ABTS—2,2-azinobis (3ehtylbenzothiazoline-6-sulfonic acid) diammonium salt; ORAC—the oxygen radical absorbance capacity; FRAP—Ferric reducing antioxidant power; DPPH—diphenyl-1-picrylhydrazyl; SOD—superoxide dismutase; TEAC—6-hydroxy-2,5,7,8-tetramethylchroman-2-carboxylic acid (Trolox)-equivalent antioxidant capacity.

## Data Availability

Not applicable.
